# Superparamagnetic Iron Oxide Nanoparticles and Static Magnetic Field Regulate Neural Stem Cell Proliferation

**DOI:** 10.3389/fncel.2021.815280

**Published:** 2022-02-04

**Authors:** Dan Li, Yangnan Hu, Hao Wei, Wei Chen, Yun Liu, Xiaoqian Yan, Lingna Guo, Menghui Liao, Bo Chen, Renjie Chai, Mingliang Tang

**Affiliations:** ^1^School of Biology, Food and Environment, Hefei University, Hefei, China; ^2^Co-innovation Center of Neuroregeneration, Nantong University, Nantong, China; ^3^School of Life Sciences and Technology, Southeast University, Nanjing, China; ^4^Department of Otorhinolaryngology Head and Neck Surgery, Drum Tower Clinical Medical College, Nanjing Medical University, Nanjing, China; ^5^Department of Otolaryngology Head and Neck Surgery, The Second Affiliated Hospital of Anhui Medical University, Hefei, China; ^6^Materials Science and Devices Institute, Suzhou University of Science and Technology, Suzhou, China; ^7^Department of Cardiovascular Surgery of the First Affiliated Hospital and Institute for Cardiovascular Science, Medical College, Soochow University, Suzhou, China

**Keywords:** SPIO, SMF, neural stem cells, proliferation, regulation

## Abstract

Neural stem cells (NSCs) transplantation is a promising approach for the treatment of various neurodegenerative diseases. Superparamagnetic iron oxide nanoparticles (SPIOs) are reported to modulate stem cell behaviors and are used for medical imaging. However, the detailed effects of SPIOs under the presence of static magnetic field (SMF) on NSCs are not well elucidated. In this study, it was found that SPIOs could enter the cells within 24 h, while they were mainly distributed in the lysosomes. SPIO exhibited good adhesion and excellent biocompatibility at concentrations below 500 μg/ml. In addition, SPIOs were able to promote NSC proliferation in the absence of SMF. In contrast, the high intensity of SMF (145 ± 10 mT) inhibited the expansion ability of NSCs. Our results demonstrate that SPIOs with SMF could promote NSC proliferation, which could have profound significance for tissue engineering and regenerative medicine for SPIO applications.

## Introduction

Neural stem cells (NSCs) act as one of the adult stem cells that are typically considered capable of giving rise to neurons and glia cell lineages (Gage, 2000). Currently, they have been widely used for spinal cord repairing and for the treatment of various neurodegenerative diseases in animal models (Gage, 2000; Madhavan and Collier, 2010; Mathieu et al., 2010; Carletti et al., 2011; Yoneyama et al., 2011; He et al., 2012; Reekmans et al., 2012). Nowadays, numerous reports have suggested that biomaterials could provide a tremendous opportunity to influence stem cell behaviors. For instance, the physicochemical properties of biomaterials, including substrate mechanical stiffness (Engler et al., 2006; Lee et al., 2013), nanometer-scale topography (Dalby et al., 2007; Yim et al., 2010; McMurray et al., 2011), and simple chemical functionality (Benoit et al., 2008; Saha et al., 2011), could regulate the stem cell fate. In particular, a previous study has shown that superparamagnetic iron oxide nanoparticles (SPIOs) promoted the proliferation of human mesenchymal stem cells (MSCs) through diminishing intracellular H_2_O_2_ and accelerating cell cycle progression (Huang et al., 2009). Furthermore, recent research has indicated that SPIOs promoted osteogenic differentiation of human bone-derived mesenchymal stem cells (hBMSCs) (Wang et al., 2016). Similarly, it has also been reported that SPIOs could promote osteogenic differentiation of adipose-derived mesenchymal stem cells (ASCs) (Xiao et al., 2015). These findings suggest that SPIOs may have the potential to modulate stem cell behaviors.

Stem cell fate is determined by the complex interactions between stem cells and their surroundings, including biochemical factors, extracellular matrix components, intercellular interactions, and physical factors (Allazetta and Lutolf, 2015). A magnetic field is an effective physical factor that has been reported to modulate cell proliferation as earlier as 1999 (Fanelli et al., 1999). Meanwhile, it was reported to mediate the osteogenic differentiation of human ASCs (Kang et al., 2013) and induce MSC differentiation into osteoblasts and cartilage (Ross et al., 2015). In addition, the magnetic fields and magnetic nanomaterials were used together to induce the growth direction of neurons (Riggio et al., 2014) and facilitate drug delivery (Qiu et al., 2017). However, there is no report to explore the biological effects of SPIO under the presence of a magnetic field on NSC behaviors.

## Materials and Methods

### Synthesis of Superparamagnetic Iron Oxide Nanoparticles

The SPIOs were coated by polyglucose-sorbitol-carboxymethylether (PSC) as modified in this experiment. The method to synthesize SPIOs was applied to the classic chemical coprecipitation combined with an excellent alternating-current magnetic field (ACMF)-induced internal-heat mode (Chen et al., 2016). Briefly, 40 mg PSC was dissolved in 2 ml of deionized water, then the mixture of the 6 mg FeCl_3_ with 3 mg FeCl_2_ dissolved in 15 ml of deionized water was added. After cooling the mixture to 5°C, 1 g 28% (w/v) ammonium hydroxide was added with stirring for over 2 min. The mixture was then heated at 80°C for 1 h, then the deionized water was purified with five cycles of ultrafiltration using a 100 kDa membrane.

### Characterization of Superparamagnetic Iron Oxide Nanoparticles

The core of synthesized SPIOs was detected by transmission electron microscopy (TEM; JEOL/JEM-200CX, Japan). The size distribution was analyzed by dynamic light scattering (DLS) with a particle size analyzer (Malvern Zetasizer Nano ZS90, United Kingdom). The hysteresis loop of SPIOs was measured using a vibrating sample magnetometer (LS 7307-9309, Lakeshore Cryotronic, United States). The final concentrations of iron in the aqueous solution were measured by inductively coupled plasma mass spectrometry (ICP-MS) on an Optima 5300DV instrument.

### Neural Stem Cell Culture

Neural stem cell isolation and culture were described in the previously published protocol [32]. Briefly, NSCs were cultured in mixed DMEM-F12 medium (Gibco, Grand Island, NY) containing 2% B-27 (Gibco), 100 U/ml penicillin, and 100 μg/ml streptomycin (Sigma-Aldrich, St. Louis, MO, United States) in the condition of 5% CO_2_ at 37°C. NSCs were passaged every 3–5 days during culturing. For the determination of NSC proliferation, cells were seeded with a concentration of 5 × 10^4^ cells/ml in DMEM-F12 medium with 2% B-27, 20 ng/ml EGF (R&D Systems, Minneapolis, MN, United States), and 20 ng/ml FGF-2 (R&D Systems, Minneapolis, MN, United States). For NSC differentiation assays, cells were seeded in an NSC differentiation kit (Stem Cell, Hangzhou, China). The care and use of animals in these experiments followed the guidelines and protocol approved by the Care and Use of Animals Committee of Southeast University. All efforts were made to minimize the number of animals used and their suffering.

### Cellular Uptake of Superparamagnetic Iron Oxide Nanoparticles by Inductively Coupled Plasma Mass Spectrometry

Cells were seeded in 25 cm^2^ flasks at the concentration of 1 × 10^5^ to 1 × 10^6^ per flask and incubated with SPIOs at the indicated concentrations. After 1, 2, 3, or 4 days of treatment, cells were harvested and counted. Then, the cell suspension was dissociated by hydrochloric acid with a final concentration of 60%. The concentration of iron in cell lysates was measured by ICP-MS according to PerkinElmer’s operating procedures.

### Cell Viability Assay

Neural stem cells were seeded in 96-well cell culture plates at the concentration of 1 × 10^5^ cells per well (*n* = 6) and cultured overnight. The cells were then incubated with differentiation concentrations of SPIOs at indicated concentrations. After culturing for 12 or 24 h, cell viability was measured by CCK-8 assay (Beyotime, Shanghai, China) according to the manufacturer’s instructions. The NSCs cultured with the ordinary medium were considered as the control.

### Immunostaining

Neural stem cells were fixed with 4% paraformaldehyde for 1 h at room temperature, following treatment with blocking medium for 1 h. Next, the cells were incubated with primary antibody diluted solution overnight at 4°C. Then, each sample was washed with phosphate buffer solution [0.1% Triton X-100 in phosphate buffer solution (PBST) twice per 5 min]. The samples were further incubated with a secondary antibody for 1 h at room temperature. Finally, samples were washed with PBST, and an antifade fluorescence mounting medium (DAKO) was added. The antibodies used were Nestin (Beyotime, China). Cell proliferation was detected by Click-it EdU imaging kit (Invitrogen). All the images were captured by a Zeiss LSM 700 confocal microscope, and ImageJ (NIH) was used for image analysis.

### Scanning Electron Microscope and Transmission Electron Microscopy Examination

Neural stem cells were seeded in 24-well cell culture plates at the concentration of 1 × 10^4^ cells per well and incubated with a differentiation concentration of SPIOs at indicated concentrations. After incubation for 3 days, cells were washed two times with 1 × PBS (pH 7.4); 2.5% glutaraldehyde solution (Alfa Aesar, Tewksbury, MA, United States) was added to each sample. Cells were co-incubated for 1 h at 37°C. Then, cells were dehydrated overnight, and the cell morphology was detected by scanning electron microscope (SEM) (Ultra Plus, Zeiss, Oberkochen, Germany).

Neural stem cells were seeded in 25 cm^2^ flasks at the concentration of 1 × 10^6^ and incubated with SPIOs at the concentration of 100 μg/ml. After 12- or 24-h treatment, the cells were harvested and washed two times with PBS; 2.5% glutaraldehyde solution was added to each sample, and cells were co-incubated overnight at 4°C to fix the cells. Then, the samples were transferred to 1% osmium tetroxide, dehydrated in ethanol, and embedded in Epon (Sigma–Aldrich). Finally, uranyl acetate and lead citrate were used for staining of ultrathin slices (60–80 nm). The images were captured by TEM (JEOL/JEM-200CX, Tokyo, Japan).

### Statistical Analysis

All data are shown as mean and SD. Statistical analyses were conducted using GraphPad Prism 6 software. For all experiments, *n* represents the number of replicates, and at least three individual experiments were conducted. One- or two-way ANOVA analysis followed by a Tukey’s *post hoc* test was used to determine the statistical significance between multiple groups, and Student’s *t*-test was used for comparisons between two groups. A value of *p* < 0.05 was considered to be statistically significant.

## Results

### Synthesis and Characterization of Superparamagnetic Iron Oxide Nanoparticles

**FIGURE 1:**
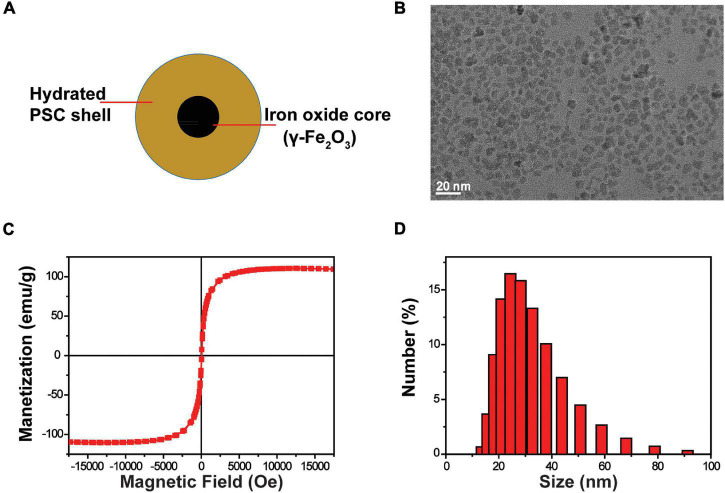
Characterization of SPIOs. **(A)** Schematic structure of SPIOs. **(B)** TEM image of SPIOs. **(C)** Hysteresis loop of SPIOs. **(D)** Particle size distributions of the SPIOs as measured by DLS.

The SPIOs were composed of a γ-Fe_2_O_3_ core and PSC shell (Figure 1A). The size of the γ-Fe_2_O_3_ core is about 6–8 nm (Figure 1B). The hysteresis loop of SPIOs is about 105 emu/g (Figure 1C), which indicates that it has a good superparamagnetic property. The average diameter of whole SPIOs measured by DLS is about 30 nm (Figure 1D). These results suggest that the SPIOs synthesized by this method have a uniform particle size, stable structure, good dispersion (PDI of 0.154), and stronger magnetic properties than the conventional coprecipitation method.

### Internalization and Cellular Uptake of Superparamagnetic Iron Oxide Nanoparticles

**FIGURE 2:**
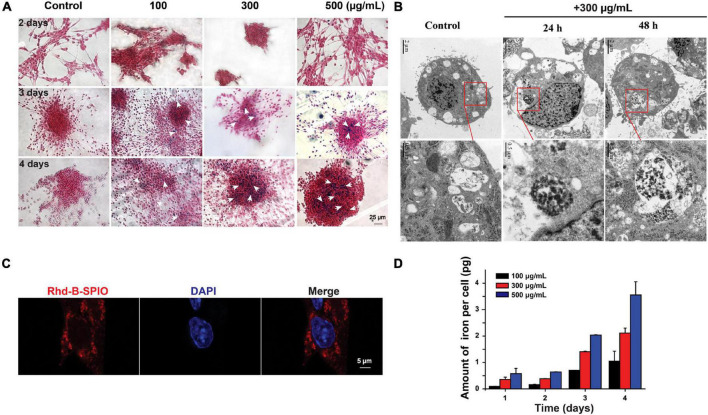
Internalization and cellular uptake of SPIOs. **(A)** Representative Perl’s blue staining images of SPIOs-treated NSCs. Scale bar = 25 μm. **(B)** TEM images of NSCs under SPIOs treatment (300 μg/ml for 24 and 48 h). A higher-magnification image of the indicated portion is shown in the inferior panel. **(C)** Laser confocal images of NSCs with Rhd-B-SPIOs treatment for 24 or 48 h. Red, Rhd-B, blue, nucleus. Scale bar = 5 μm. **(D)** The amount of intracellular iron uptake in NSCs after SPIOs treatment for different concentrations at the indicated time.

Superparamagnetic iron oxide could enter the cells after co-incubation for 1 day (Figure 2A). The amount of SPIO could increase with time and concentrations of SPIO (Figure 2D). It was noticed that there was no significant difference between the 3- and 4-day groups, indicating that the uptake by a single cell was stale within 3 days. Furthermore, TEM and fluorescence imaging were employed to examine the internalization of SPIOs in NSCs. TEM results revealed that SPIOs were located in the lysosomes after 24 and 48 h of exposure (Figure 2B). It was further verified that SPIOs were located outside the cell nucleus after 24 h of exposure (Figure 2C).

**FIGURE 3:**
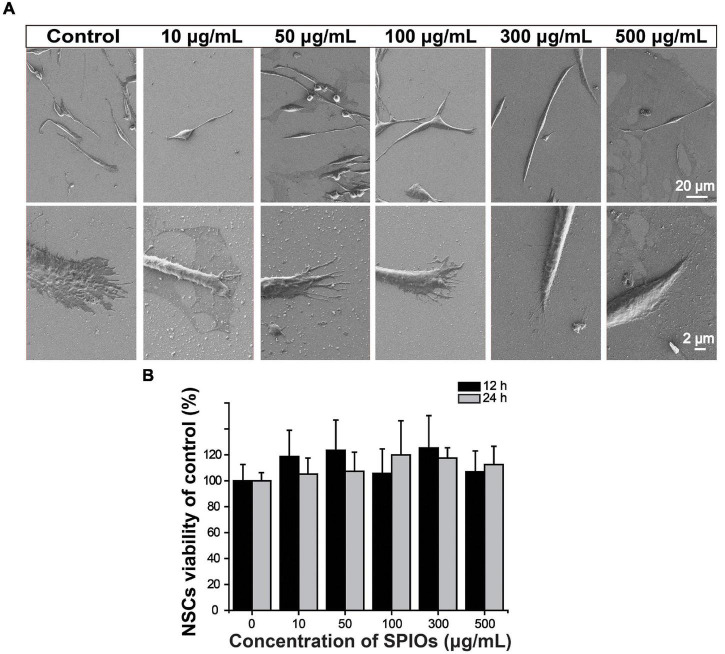
Biocompatibility of SPIOs. **(A)** SEM images of NSCs after culturing with SPIO for 3 days at indicated concentrations. **(B)** Cell viability was detected by the CCK-8 assay. The cells were treated with SPIOs for 12–24 h at indicated concentrations. Data were normalized to the control group (no SPIOs exposure). Data are presented as mean ± SD. Student’s *t*-test.

The morphology of NSCs was observed by SEM after 3-day co-incubation with various concentrations of SPIOs. Cells in all groups presented normal phenotypes (Figure 3A). Importantly, SPIOs exposure did not affect NSC viability at concentrations up to 500 μg/ml for 24 h (Figure 3B), indicating the good biocompatibility of SPIOs.

### Effects of Superparamagnetic Iron Oxide Nanoparticle and Static Magnetic Field on Neural Stem Cell Proliferation

**FIGURE 4:**
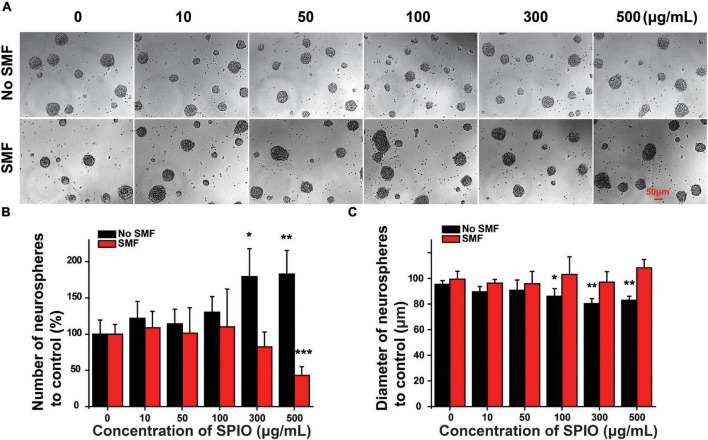
The number and diameter of neurospheres. NSCs were cultured with indicated concentrations of SPIO (10, 50, 100, 300, and 500 μg/ml) with or without SMF for 3 days. **(A)** Representative optical images of neurospheres. **(B)** Quantification of the neurospheres number. **(C)** Quantification of the neurospheres diameter in the experimental groups. Data are presented as mean ± SD, *p < 0.05, **p < 0.01, and ***p < 0.001.

Neural stem cell proliferation was first evaluated by neurosphere formation assay. NSCs from higher concentrations of SPIOs (300 and 500 μg/ml) had a significantly higher rate of neurosphere formation compared to the control group, while lower concentrations of SPIOs (10, 50, and 100 μg/ml) or 50 ± 10 mT SMF treatment had no obvious effect on the number of neurosphere formation (Figures 4A,B). In contrast, SPIOs at different concentrations (100, 300, and 500 μg/ml) simultaneously exposed to SMF (50 ± 10 mT) resulted in a slower rate of neurosphere formation (Figures 4A,B). Although more neurospheres were generated from NSCs treated with higher concentration SPIOs, there was no significant effect on the diameters of the neurospheres compared to the control group (Figures 4A,C). Notably, when the NSCs were simultaneously exposed to SMF, the neurospheres exhibited a larger diameter at concentrations of 100–500 μg/ml.

**FIGURE 5:**
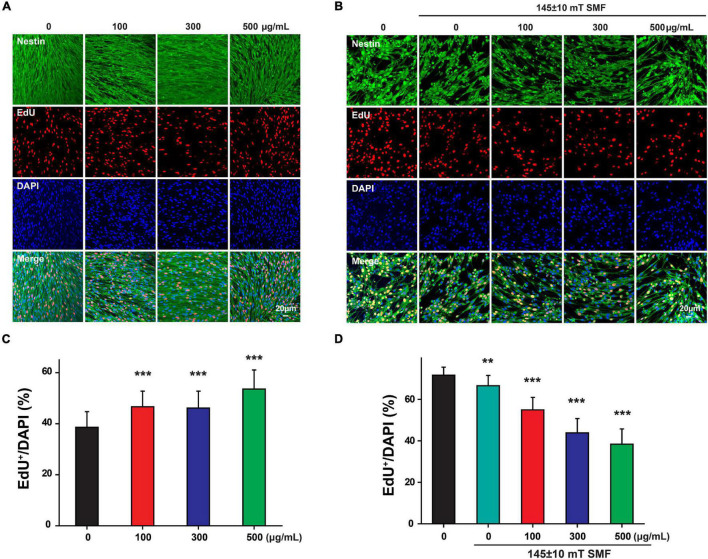
Neural stem cells proliferation was measured by EdU labeling. NSCs were cultured with indicated concentrations of SPIO (100, 300, and 500 μg/ml) with or without 145 ± 10 SMF for 3 days. EdU was added to the culture from day 2 to day 3. Representative images for EdU staining in **(A)** control group and **(B)** 145 ± 10 mT SMF group with or without SPIO treatment at indicated concentrations (100, 300, and 500 μg/ml). The ratio of EdU + /DAPI was shown in **(C,D)**, respectively. Data are presented as mean ± SD, **p < 0.01, ***p < 0.001.

Since the low concentration of SPIOs (10 and 50 μg/ml) had no effect on NSC proliferation regardless of SMF presence, these two concentrations were not included in the subsequent experiments. Next, EdU^+^/DAPI cells were counted to further examine NSC proliferation. NSCs treated with 100, 300, and 500 μg/ml SPIOs generated significantly more EdU^+^/DAPI cells than those from the control group (control group: 38.60 ± 6.11%; 100 μg/ml: 46.61 ± 6.16; 300 μg/ml: 46.13 ± 6.62; 500 μg/ml: 53.57 ± 7.49%; *p* < 0.001) (Figures 5A,C). Furthermore, SMF (145 ± 10 mT) presence inhibited the ratio of EdU^+^/DAPI cells when compared to the control (0 μg/ml: 71.62 ± 3.93%; SMF: 66.58 ± 4.98%; *p* < 0.001). Next, when NSCs were cultured in the presence of SPIOs (100, 300, and 500 μg/ml) plus SMF (145 ± 10 mT) for 3 days, significantly lower ratio of EdU^+^/DAPI cells was observed (100 μg/ml: 54.92 ± 6.03%; 300 μg/ml: 43.79 ± 6.93%; 500 μg/ml: 38.37 ± 7.39%) (Figures 5B,D).

**FIGURE 6:**
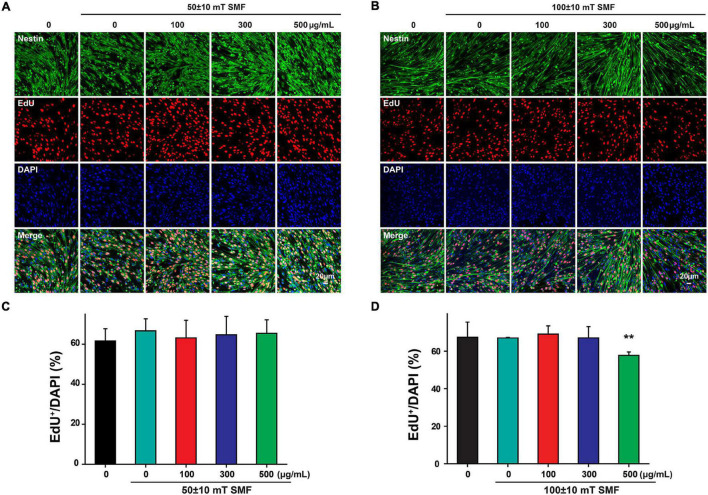
Neural stem cells proliferation was measured by EdU labeling. NSCs were cultured with indicated concentrations of SPIO (100, 300, and 500 μg/ml) with or without SMF for 3 days. EdU was added to the culture from day 2 to day 3. Representative images for EdU staining in **(A)** 50 ± 10 mT group and **(B)** 100 ± 10 mT SMF group with or without SPIO treatment at indicated concentrations (100, 300, and 500 μg/ml). The ratio of EdU + /DAPI was shown in **(C,D)**, respectively. Data are presented as mean ± SD, **p < 0.01.

Interestingly, when the SMF intensity was reduced to 50 ± 10 mT, the exposure of SPIOs at concentrations of 100–300 μg/ml failed to reduce the ratio of EdU^+^/DAPI cells (Figures 6A,C). However, 100 ± 10 mT SMF could significantly suppress NSC proliferation when SPIO concentration was more than 500 μg/ml, as evidenced by the decreased ratio of EdU^+^/DAPI cells (Figures 6B,D).

## Discussion

Stem cells have a wide application prospect in the biomedical fields. NSCs have been verified for their potential in the treatment of various diseases, especially neural diseases [3–9]. The stem cell niche is the interaction between stem cells and their microenvironment which is regarded as key players in stem cell fate decisions. The niche includes several physical factors, biochemical factors, and extracellular matrix components (Allazetta and Lutolf, 2015). Many biomaterials have been proposed to modulate the stem cell niche to further regulate stem cell fate. For example, SPIOs have been reported to be able to modulate stem cell behaviors, including proliferation and differentiation (Huang et al., 2009; Xiao et al., 2015; Wang et al., 2016). Meanwhile, the magnetic field is also confirmed as one of the physical factors that affect stem cell fate decisions. In this research, we introduced SPIOs and magnetic fields together to explore whether they could affect NSC proliferation.

Some types of SPIOs have been reported to exert excellent biocompatibility, while the potential toxicity under certain conditions (e.g., surface modification) is still under debate. Numerous studies focus on SPIO cytotoxicity on different types of cells. SPIO labeling was found not to alter MSC viability and apoptosis (Schafer et al., 2009; Zhang et al., 2014; Singh et al., 2019; Xu et al., 2020). It was further revealed that SPIOs coated with unfractionated heparin did not affect MSC survival (Lee et al., 2012). Furthermore, histological examination showed that silica-coated SPIOs induced no obvious tissue impairments or abnormal inflammation and pathology in major organs (Ledda et al., 2020). Our results were consistent with the above reports that SPIOs at concentrations less than 500 μg/ml did not affect NSC adhesion or induce cell death. SPIO toxicity is believed to be highly correlated with the cores and coatings. Usually, the SPIO core should have a magnetic responsive component. Some high-magnetic materials such as nickel are easy to be oxidized, thus leading to certain toxicity (Tartaj and Serna, 2003; Mahmoudi et al., 2011). The main iron oxides, hematite (α-Fe_2_O_3_), maghemite (γ-Fe_2_O_3_), and magnetite (Fe_3_O_4_) are superparamagnetic and also have good biocompatibility. In this study, SPIOs were prepared by an alternating-current magnetic field (ACMF)-induced internal-heat mode that was described previously (Chen et al., 2016). The SPIOs synthesized in this study were composed of a γ-Fe_2_O_3_ core and PSC shell. This type of SPIOs has good biosafety, as evidenced by our results. In addition, size could be a critical factor to determine SPIO cytotoxicity. It is believed that SPIOs with a diameter ranging from 10 to 100 nm are considered to be optimal for the purpose of systemic administration (Wahajuddin and Arora, 2012). SPIO toxicity was also reported with a particle size within this range. In this study, the SPIO size was within 20–40 nm, and no obvious cytotoxicity was observed at this range.

In summary, we found that SPIOs are a potential regulator for NSC expansion. SPIOs at appropriate concentrations can elevate the proliferation ability of NSCs. In the meantime, our results also indicate that SMF may suppress NSC proliferation at high intensity. In the future, we will make the best effort to uncover the biological effects of SPIOs on NSC behaviors, including migration and differentiation. Also, the detailed mechanisms underlying the observed effects will be explored as well.

## Data Availability Statement

The raw data supporting the conclusions of this article will be made available by the authors, without undue reservation.

## Ethics Statement

The animal study was reviewed and approved by Southeast University.

## Author Contributions

DL, YH, HW, WC, YL, XY, LG, ML, and BC conducted the experiments and analyzed the data. RC and MT supervised the study and acquired the funding. All authors wrote the manuscript.

## Conflict of Interest

The authors declare that the research was conducted in the absence of any commercial or financial relationships that could be construed as a potential conflict of interest. The reviewer FY declared a shared affiliation, with several of the authors YH, WC, YL, XY, ML, and RC to the handling editor at the time of the review.

## Publisher’s Note

All claims expressed in this article are solely those of the authors and do not necessarily represent those of their affiliated organizations, or those of the publisher, the editors and the reviewers. Any product that may be evaluated in this article, or claim that may be made by its manufacturer, is not guaranteed or endorsed by the publisher.
